# Alcohol and Cardiovascular Disease: Helpful or Hurtful

**DOI:** 10.31083/j.rcm2404121

**Published:** 2023-04-19

**Authors:** Akash Patel, Vincent M. Figueredo

**Affiliations:** ^1^Department of Medicine, St Mary Medical Center, Langhorne, PA 19047, USA; ^2^Department of Cardiology, St Mary Medical Center, Langhorne, PA 19047, USA

**Keywords:** cardiovascular, vascular function, alcohol abuse, stroke, cardiomyopathy

## Abstract

Alcohol has been considered throughout history as both a tonic and a poison. The answer as to which likely depends on one’s current health, the amount one consumes, and with what regularity. In examining the relationship of alcohol and cardiovascular health, most, but not all, epidemiological studies suggest that light to moderate alcohol consumption can reduce the incidence of coronary artery disease (CAD), ischemic stroke, and peripheral arterial disease events. Conversely, abuse of alcohol can lead to cardiomyopathy, heart failure, sudden death, and hemorrhagic strokes. In this article, we review the literature studying the effects of alcohol on coronary artery disease and stroke. A recently published study concluded there was no amount of alcohol per day that was heart healthy. Yet more than one hundred previous studies have found that people who drink in moderation have a lower risk of cardiovascular disease events when compared to those who do not drink or drink heavily. Moderate drinking is defined as one to two drinks per day; where one drink is defined as 12 ounces of beer, 5 ounces of wine or 1.5 ounces of hard liquor. In this article we reviewed the data suggesting that consuming alcohol in moderation on a regular basis–as opposed to 7 drinks on Saturday night–could have cardiovascular protective effects.

## 1. Introduction

Alcohol has been considered throughout history as both a tonic and a poison. The answer as to which likely depends on one’s current health, the amount one consumes, and with what regularity. In examining the relationship of alcohol and cardiovascular health, most, but not all, epidemiological studies suggest that light to moderate alcohol consumption can reduce the incidence of coronary artery disease (CAD), ischemic stroke, and peripheral arterial disease events. Conversely, abuse of alcohol can lead to cardiomyopathy, heart failure, sudden death, and hemorrhagic strokes. In this article, we reviewed the recent literature on PubMed to update past reviews of the association between alcohol and cardiovascular disease.

A recent study concluded there was no amount of alcohol per day that was heart healthy [1]. Yet more than one hundred previous studies have found that people who drink in moderation have a lower risk of cardiovascular disease events when compared to those who do not drink or drink heavily [2, 3, 4, 5, 6, 7, 8, 9, 10]. Moderate drinking is defined as one to two drinks per day; where one drink is defined as 12 ounces of beer, 5 ounces of wine or 1.5 ounces of hard liquor [4]. In this article we reviewed the data suggesting that consuming alcohol in moderation on a regular basis–as opposed to 7 drinks on Saturday night–may be associated with cardiovascular protective effects.

## 2. Alcohol and Coronary Artery Disease

Coronary artery disease (CAD) is one of the most significant health burdens in modern societies. CAD causes higher numbers of death and loss of Disability Adjusted Life Years (DALYs) compared to all other diseases [2]. The number of deaths due to CAD has increased by 50% since the 1990s. CAD is the leading cause of death in the United States, and the third leading cause around the world [3]. Significant consumption of alcohol has been shown to be associated with development of CAD [4]. Conversely, some studies have found cardiovascular beneficial effects of alcohol in moderation [4].

The role of alcohol in CAD has been argued for decades by experts and researchers. The amount of alcohol consumed has been shown to be a major factor determining the potential beneficial or harmful effects for CAD; a similar phenomenon being observed in the relationship of alcohol to other cardiovascular diseases [4]. In 1986, Moore and Pearson, reviewed cross-sectional, cohort, and epidemiological studies across various populations examining the relationship between moderate alcohol consumption and CAD, and concluded that there is an inverse relationship between moderate alcohol use and CAD development. The findings were attributable to the beneficial effects of moderate use of alcohol on high-density lipoprotein (HDL) and apoliporoteins [5]. Similarly, another more recent review came to a similar conclusion citing more evidence [6]. Furthermore, the association of moderate alcohol use and lower risk of CAD has been strengthened by findings in a number of meta-analyses over the past decade. Costanzo *et al*. [7], in their meta-analysis found 30% more protection against vascular risk, including CAD, in patients who consumed 20 g of alcohol per day. Similarly, Zhang *et al*. [8] pooled data from 35 studies comparing moderate drinkers with non-drinkers and found a reduced risk of CAD in moderate drinkers, with an odds ratio (OR) of 0.68. Yet another meta-analysis, involving nearly 100,000 participants and 39,000 ischemic heart disease events, also found a cardioprotective association with low to moderate alcohol use for ischemic heart disease [9]. Song and colleagues, in a study on US veterans found that low to moderate alcohol consumption reduced the risk of CAD in veterans [10]. Regardless of population studied, sex, or type of alcohol consumed in regular moderation, the relationship between alcohol intake and ischemic heart disease demonstrated a J-shaped relationship [2, 3, 4, 5, 6, 7, 8, 9, 10]. Low to moderate alcohol consumption appeared to have a protective effect against coronary artery disease events when compared to heavy alcohol consumption or abstention.

Some have argued that the inverse association between moderate alcohol consumption and CHD may be partially attributed to a poorly defined reference group. For example, the reference group of non-drinkers could include heavy drinkers who deny alcohol intake or who have stopped drinking alcohol due to illness. More recent studies are better at excluding individuals who abstain for health reasons and have found a similar reduction in risk, suggesting that the reduction is not attributed to this potential bias.

In contrast to the aforementioned studies and meta-analyses, Biddinger *et al*. [1] recently concluded there was no amount of alcohol per day that was healthy. However, when reviewing the data from the study, we and others [11] find that light alcohol intake (3–6 drinks per week) shows cardiovascular benefits.

In contrast to the beneficial effects of moderate use, excess alcohol consumption has been found to be harmful, increasing the risk for CAD [12, 13, 14]. Studies have suggested that light to moderate drinkers who also partake in episodic binge drinking, have increased risk of CAD [13, 15]. In contrast to numerous studies finding that binging has no cardioprotective effect, and possibly a negative one, a recent study by Degerud *et al*. [16] did not find a significant association between binge drinking and ischemic heart disease. Table 1 (Ref. [16, 17, 18, 19, 20, 21, 22, 23, 24, 25, 26, 27, 28, 29, 30, 31, 32, 33, 34, 35, 36, 37, 38, 39]) shows study characteristics for studies included in the above reviews and meta-analyses examining moderate alcohol use and coronary artery disease.

**Table 1.** **Study Characteristics for Studies Included in Reviews and Meta-Analyses Reported on Examining Moderate Alcohol Use and Coronary Artery Disease**.

Study	Study design	Country	Number of participants	% Men and women	Average age (years)	Alcohol consumption	HR/OR/RR (95% Confidence Interval)
Yusuf *et al*., 2004 [17]	Case Control	52 countries (North America, Asia, Europe, Middle East, Africa)	15,463	80.6% Men	61.5	3 or more drinks/week	OR: 0.91 (0.82–1.02)
				19.4% Women			
Hvidtfeldt *et al*., 2010 [18]	Prospective	North America, Europe	23,733	47.9% Men	52.5	15–29.9 g/day	HR: Men 0.72 (0.64–0.82)
				52.1% Women			Women 0.52 (0.40–0.67)
Mukamal *et al*., 2006 [19]	Prospective	United States of America	8867	100% Men	56.0	15–29.9 g/day	HR: 0.38 (0.16–0.89)
Ikehara *et al*., 2013 [20]	Prospective	Japan	47,100	100% Women	49.3	20–45 g/day	HR: 0.84 (0.36–1.97)
Arriola *et al*., 2009 [21]	Prospective	Spain	41,438	37.8% Men	49.0	5–30 g/day	HR: Men 0.49 (0.32–0.75)
				62.2% Women			Woman 0.62 (0.36–1.07)
Tolstrup *et al*., 2006 [22]	Prospective cohort	Denmark	53,500	46.7% Men	50–65 years	24–48 g/week	HR: Men 0.78 (0.66–0.94)
				53.3% Women			Women 0.63 (0.52–0.77)
Britton and Marmot, 2004 [23]	Prospective cohort	United Kingdom	10,308	100% Men	35–55 years	10–80 g/week	HR: Men 0.99 (0.78–1.25)
							Women 0.96 (0.67–1.38)
Athyros *et al*., 2007 [24]	Cross-Sectional	Greece	4153	49.0% Men	47.4	20–45 g/day	OR: Men 0.62 (0.49–0.82)
				51.0% Women			Women 0.57 (0.32–0.76)
Tanasescu *et al*., 2001 [25]	Prospective	United States of America	2419	100% Men	60.1	0.5–2 drinks/day (7–28 g/day)	RR: 0.64 (0.40–1.02)
Kitamura *et al*., 1998 [26]	Prospective	Japan	8476	100% Men	40–59 years	23–45 g/day	RR: 0.55 (0.29–1.05)
Rimm *et al*., 1991 [27]	Prospective	United States of America	44,059	100% Men	53.1	15–30 g/day	RR: 0.73 (0.51–1.05)
Solomon *et al*., 2000 [28]	Prospective cohort	United States of America	5103	100% Women	48.1	0.1–4.9 g/day	RR: 0.45 (0.29–0.68)
Mukamal *et al*., 2003 [29]	Prospective	United States of America	5882	100% Men	53.6	15–29.9 g/day	RR: 0.79 (0.64–0.96)
Beulens *et al*., 2007 [30]	Prospective cohort	United States of America	11,711	100% Men	60.0	15–29.9 g/day	HR: 0.72 (0.54–0.97)
Bos *et al*., 2010 [31]	Prospective cohort	Europe	17,357	100% Women	54.0	30–69.9 g/day	HR: 0.92 (0.68–1.24)
Fernandez-Jarne *et al*., 2003 [32]	Case control	Spain	171	96% Men	59.5	20–29.9 g/day	OR: 0.41 (0.12–1.34)
				4% Women			
Fuchs, 2004 [33]	Prospective cohort	United States of America	14,506	33.2% White Men	45–64 years	140–210 g/week for Men	HR: White men 0.81 (0.45–1.44)
				39.9% White Women		1–70 g/week for Women	White women 0.64 (0.36–1.12)
				10.0% Black Men			Black men 2.67 (1.12–6.34)
				16.8%Black Women			Black women 0.49 (0.20–1.18)
Degerud *et al*., 2021 [16]	Cross-sectional	Norway	44,476	70.7% Men	49.7	12–23.99 g/day	HR: 0.82 (0.58–1.17)
				29.9% Women			
Ebbert *et al*., 2005 [34]	Prospective cohort	United States of America	30,518	100% Women	61.3	1–14 g/day	HR: 0.77 (0.61–0.97)
Murray *et al*., 2002 [35]	Prospective cohort	Canada	1154	50.3% Men	42.0	5.78–18.1 g/day for Men	HR: Men 0.70 (0.26–1.85)
				49.7% Women		2.93–9.15 g/day for Women	Women 0.96 (0.67–1.38)
Song *et al*., 2017 [36]	Observational cohort	United States of America	156,728	95% Men	67.3	12–24 g/day	HR: 0.79 (0.61–0.81)
				5% Women			
Mukamal *et al*., 2006 [37]	Prospective	United States of America	4410	54.8% Men	72.4	7–13 drinks/week (84–156 g/week)	RR: 0.71 (0.50–1.01)
				45.2% Women			
Ikehara *et al*., 2009.[38]	Prospective	Japan	19,356	100% Men	53.1	150–299 g/week	HR: 0.23 (0.12–0.37)
Marques-Vidal *et al*., 2004 [39]	Prospective	France, Ireland	7352	100% Men	54.8	7–14 drinks/week (84–168 g/week)	RR: 0.65 (0.39–1.08)

OR, odds ratio; HR, hazard ratio; RR, relative risk.

An often-heard criticism of this theory of alcohol-mediated cardioprotection is that investigators receive funding or are influenced by the alcohol industry to find this “positive” association. While early studies may have been unduly influenced, investigators are becoming increasingly aware of the issues related to conflict of interest. We reviewed the funding sources for all studies referenced. All were funded by their country or university, with a majority funded by the US National Institutes of Health (NIH). Only one study [5] was funded by the Alcoholic Beverage Research Foundation, as well as the NIH. Furthermore, there are multiple plausible mechanisms of alcohol’s cardioprotective effect that have been demonstrated.

## 3. Possible Mechanism(s) of Alcohol’s Cardioprotective Effect

The exact mechanism(s) of the beneficial effects of moderate drinking on CAD are not completely understood, but several hypotheses have been put forward. Alcohol favorably effects cardiovascular biomarkers, which in turn decreases risk of CAD. For example, moderate consumption increases HDL cholesterol and adiponectin, and decreases fibrinogen [40]. Alcohol can also indirectly lower risk of CAD by affecting risk factors. For example, studies have shown that low to moderate alcohol use positively affects insulin sensitivity, protecting against diabetes, and in turn CAD [41].

Alcohol can affect the constriction and relaxation of blood vessels. Studies have reported that heavy ethanol hinders the nitric-oxide generating system of endothelium [42]. In contrast, low to intermediate ethanol improves the endothelial function by increasing the expression of nitric oxide synthases. For example, Liu *et al*. [43] found that low ethanol improved endothelial nitric oxide synthase (eNOS) expression in endothelial cells *in vitro*. Similar results were reported by Kleinhenz *et al*. [44]. On the other hand, higher amounts of ethanol disrupted the endothelial function by reducing the production of eNOS [45]. In a case control study, researchers found that heavy alcohol consumers had impaired endothelial vasodilation compared to normal subjects [46]. Binge alcohol drinking also disrupts endothelial function as shown by Hijmering *et al*. [47]. They found that binge alcohol consumption caused endothelial dysfunction by decreasing flow mediated vasodilation. Another study found that previous heavy alcoholics, even after abstinence, had persistent endothelial dysfunction [48].

A study by Whitfield *et al*. [49] reported that HDL cholesterol increases as alcohol amount is increased, while there is an inverse relationship with insulin levels. There was a J-shaped relationship between the amount of alcohol consumed and triglycerides levels. Xiao *et al*. [50], in a cross-sectional study involving 20,502 participants, reported increases in HDL cholesterol and decreasing triglycerides associated with alcohol consumption. In another study, Budzyński and colleagues [51] studying the effect of alcohol abstinence on plasma lipid levels, found a statistically significant increase in plasma low-density lipoprotein (LDL) and decrease in HDL and lipoprotein after 4 weeks of alcohol discontinuation. A cross-sectional study involving 4850 participants aged 65 years and older investigated the effect of alcohol on lipoprotein sub particles. They found that use of alcohol was associated with low levels of total LDL, small LDL, very-low-density lipoprotein (VLDL), and small HDL particles while there was increase in large and medium HDL particles [52]. These effects of alcohol were also supported by studies using Mendelian Randomization approach. For example, Tabara *et al*. [53], reported increased HDL and decreased LDL and triglycerides with the use of alcohol.

## 4. Alcohol and Cerebrovascular Disease

According to the data from the World Stroke Organization, 13 million individuals suffer a stroke each year. Sixty percent of these cases occur in individuals under age 70, and 8% of these occur in individuals under age 44. Stroke is the third leading cause for death and disability worldwide [54]. Alcohol use is considered as an important risk factor for stroke.

According to the data from the World Stroke Organization, 66% of strokes are attributable to behavioral and lifestyle factors. Alcohol use contributed to 11% of that burden [54]. Many studies in the past have reported a positive link between heavy alcohol use and cerebrovascular disease. Klatsky *et al*. [55] found that heavy drinking was associated with increased risk of hemorrhagic stroke. Other studies have found a relationship of alcohol use with the development of aneurysmal subarachnoid hemorrhage [56, 57] and hemorrhagic stroke [58, 59]. In contrast, studies have also found that alcohol consumption in moderate quantities can provide a protective effect [60].

A study conducted by Jimenez at all reported a U-shaped relationship between the use of alcohol and risk of stroke in women [61]. A Japanese study, investigating the association of light to moderate alcohol and risk of stroke, involving 19,550 individuals with follow up of 10 years, found that light to moderate use does not increase the risk of stroke [62]. A recent meta-analysis published in 2016, including articles from 1966–2016, sought to determine the association of alcohol consumption with different stroke types. The analysis showed that low to moderate consumption was associated with lower risk of stroke development and higher consumption was associated with higher risk of stroke. More specifically, light and moderate alcohol consumption was shown to reduce the risk of ischemic stroke, but not hemorrhagic stroke, whereas heavy use increased the risk of both ischemic and hemorrhagic stroke [63]. Another meta-analysis, investigating the relationship between alcohol use and morbidity and mortality due to various types of strokes, reported that light to moderate consumption may be protective, while excessive use increases the risk of stroke. They found a J-shaped association between alcohol use and different types of strokes [64]. A recent study Zhang and colleagues reported a J-shaped association with alcohol use and stroke in the Chinese population. A lower quantity of alcohol (1–150 g/wk) reduced the risk, while excessive intake (>350 g/wk) increased the risk of stroke [65]. In contrast, another larger prospective study involving 512,715 Chinese adults and followed for ten years did not find a protective effect of alcohol for stroke. Instead they found a positive log-linear association of stroke risk with alcohol consumption [66].

## 5. Conclusions

Current guidelines, and most physicians today, will recommend to their patients limiting alcohol intake to one drink per day for women, and two drinks per day for men. Favorable biological effects include anti-inflammatory, antioxidant, and hemorheological effects, as well as modulation of cardiovascular risk factors. Epidemiological data suggest that alcohol in moderation is associated with cardiovascular protective effects. While laboratory studies on the effects of alcohol on cardiovascular disease risk factors suggest mechanisms of alcohol-mediated cardioprotection, clinical studies to date can only suggest clinical benefit and are hypothesis generating. Randomized control trials will be required to further clarify whether such protection is clinically found.

Due to the lack of randomized control trials, routine recommendations to initiate alcohol consumption for cardioprotection should not be made, especially given its potential for addiction and abuse. Nevertheless, from our review of the current literature there is no significant justification to encourage abstinence in light to moderate drinkers.

We reviewed the funding sources from the studies reported in this review and saw little funding from the alcohol industry. However, we cannot know whether funding sources from the alcohol industry may have not been listed by all authors. The question of industry influence must be acknowledged and has been studied [67, 68, 69].

Most will agree that if you are physically active, do not smoke, eat a healthy diet, are not overweight, and have no family history of heart disease, there is little added benefit from drinking alcohol to protect your heart. However, in patients at risk for cardiovascular events who already responsibly consume alcohol, there is little reason to encourage abstinence.
